# The prediction of T- and B-combined epitope and tertiary structure of the Eg95 antigen of *Echinococcus granulosus*

**DOI:** 10.3892/etm.2013.1187

**Published:** 2013-06-28

**Authors:** XIUMIN MA, XIAOTAO ZHOU, YUEJIE ZHU, YANHUA LI, HONGYING WANG, WULAMU MAMUTI, YUJIAO LI, HAO WEN, JIANBING DING

**Affiliations:** 1 State Key Laboratory Incubation Base of Major Diseases in Xinjiang and Xinjiang Key Laboratory of Echinococcosis, First Affiliated Hospital of Xinjiang Medical University, Urumqi, Xinjiang 830000, P.R. China; 2 College of Basic Medicine of Xinjiang Medical University, Urumqi, Xinjiang 830000, P.R. China; 3 National Clinical Research Base of Traditional Chinese Medicine of Xinjiang Medical University, Urumqi, Xinjiang 830000, P.R. China

**Keywords:** Eg95, tertiary structure, T- and B-combined epitope

## Abstract

Echinococcosis, also known as hydatid disease, is a type of zoonotic parasitic disease caused by the *Echinococcus* larvae infection. The disease is severely harmful to both humans and animals. Research and development of an epitope vaccine is crucial. To determine the dominant epitopes of the Eg95 antigen, the tertiary structure and the T- and B-combined epitope of the Eg95 protein for *Echinococcus granulosus* were predicted and analyzed in the present study. The tertiary structure of the Eg95 protein was predicted using the 3DLigandsite server and RasMol software. The T- and B-combined epitope of the Eg95 antigen was analyzed using the DNAStar (V5.0), IEDB, SYFPEITHI and BIMAS. Tertiary structure prediction results showed that there were potential epitopes in Eg95 antigen. Bioinformatics analysis revealed the T- and B-combined epitopes of Eg95 antigen. Four and six T- and B-combined epitopes induced immune responses in humans and mice. Additionally, four T- and B-combined epitopes induced immune responses in both humans and mice. The tertiary structure and T- and B-combined epitopes of the Eg95 protein were also determined. The results obtained in the present study may be beneficial in the investigation of Eg95 antigenicity and the development of dominant epitope vaccines.

## Introduction

Echinococcosis, also known as hydatid disease, is a type of zoonotic parasitic disease caused by the *Echinococcus* larvae infection. This disease is severely harmful to both human and animal health. The disease has a worldwide distribution, being identified in Asia, Africa, Europe, and North America (1,2). The two types of *Echinococcus* that may cause echinococcosis in humans are *Echinococcosis granulosu*s (Eg) and *Echinococcus multilocularis* (Em). A high incidence of hydatid disease has been identified in China, with primary hydatid disease being identified in 25 regions, including provinces, municipalities and autonomous regions (3). The disease is predominantly endemic in pasture areas including Xinjiang, Qinghai, Gansu and Ningxia and semi-pasture areas. According to the incomplete statistics obtained for a number of provinces experiencing an epidemic of this disease, >50 million people were threatened by *Echinococcus granulosus* infection and ~50–60 million people were treated for echinococcosis (4). Current treatments for echinococcosis including preventive measures and surgery in combination with chemotherapy are ineffective. Thus efforts have been made to develop more effective prevention and treatment measures. Consequently, with the development of molecular biotechnology, molecular vaccine prevention against echinococcosis has become an ideal method of treating hydatid disease (5,6).

The research and development involved in identifying an epitope vaccine is an extremely difficult but highly targeted technology which comprehensively utilizes the technology of molecular biology and immunology. A key step in the preparation of the epitope vaccine involves the identification of and obtaining data pertaining to the epitope. Traditional epitope screening methods, including mass spectrum technology, nuclear magnetic resonance technology and the enzymatic method, are time-consuming, laborious and costly contributing to the low feasibility of these methods. Advances in technology has resulted in epitope prediction with bioinformatics technology becoming a more convenient and viable method. Prediction using various parameters and methods has greately improved the accuracy of epitope prediction. The epitope peptide vaccine has also been used in immune prevention against viruses (7,8). The epitope vaccine is a synthesized polypeptide based on the information of B- and T-cell epitopes. Utilization of these vaccines may protect the body against specific pathogens by activating B cells to produce specific antibodies. These vaccines potentially activate the cytotoxic T lymphocytes (CD8^+^ T cells) to eliminate virus-infected cells (9,10). In addition, CD4^+^ T cells may be activated to mediate humoral immune response.

The wide application of immunology, genetic engineering, protein engineering, synthetic peptide technology, peptide library technology, biophysical techniques, immunoassay technology and computer technology have resulted in the identification of additional antigen epitopes. Lightowlers *et al*(11,12) demonstrated that sheep (intermediate host) immunized with the Eg95 recombinant protein vaccine were protected against oncosphere infection. The immune protective effect was ≤95–100%, of which 86% were fully protected. These data suggest that Eg95 is an ideal protective antigen. In a previous study (13), *Eg95* gene was identified as a candidate gene in *Echinococcus* with high antigenicity. Additionally, the secondary structure of Eg95 had many random coils, which indicated a high flexibility. The regions with a high flexibility were potential epitope regions. Previously, we also predicted the T- and B-cell epitopes of Eg95. In this study, in order to examine the antigenicity of Eg95, we initially predicted its tertiary structure. Based on the T/B epitope information obtained as well as the tertiary structure, the T- and B-combined epitopes of Eg95 were analyzed. The results of the present study therefore provided additional evidence on the epitope information of Eg95.

## Materials and methods

### Reagents and materials

TRIzol was purchased from Invitrogen (Carlsbad, CA, USA). DL2000 DNA marker, λ *Hind*III, *Bam*III, *Sac*I, *Not*I, *Taq* enzyme and T4 DNA ligase were purchased from Takara Bio, Inc. (Shiga, Japan). AMV First Strand cDNA Synthesis kit and pUCm-T were purchased from MBI. UNIQ-10 mini plasmid extraction kit, UNIQ-10 column DNA gel extraction kit, X-gal, IPTG, LB medium (liquid and solid) and ampicillin were purchased from Sangon, Shanghai, China.

*E. coli* DH5α was provided by the Echinococcosis Institute of the First Affiliated Hospital of Xinjiang Medical University.

### Echinococcus protoscolex collection

*Echinococcus* protoscolex were collected from fresh goat liver infected with *Echinococcus*. Briefly, *Echinococcus* cyst fluid was taken with a sterile syringe and placed in a sterile beaker. The protoscolex were then allowed to naturally precipitate. After rinsing three times with sterile saline the collected protoscolex were stored at −80°C prior to analysis.

### Reverse-transcribed PCR (RT-PCR)

*Echinococcus* protoscolex were ground in liquid nitrogen prior to RNA extraction. Total RNAs were extracted by TRIzol reagents according to the manufacturer’s instructions and dissolved in 50 μl DEPC water. Then, 5 μl were run on a 1.2% MOPS-formaldehyde denaturing gel. Eg95 cDNA was reversed transcribed using the AMV of First Strand cDNA Synthesis kit following the manufacturer’s instructions. Briefly, 1 μl of total RNA was reverse transcribed into cDNA in 20 μl reaction mixtures containing 200 units of Moloney murine leukemia virus reverse transcriptase, 1 μl per reaction of oligo(dT)18 primers, and 0.5 mM each of dNTPs, dATP, dCTP, dGTP, and dTTP. The reaction mixture was then incubated at 42°C for 1 h and at 70°C for 5 min to deactivate the reverse transcriptase.

### Cloning of Eg95 gene by PCR

The *Eg95* gene was cloned using the protoscolex cDNA. The primer pairs were designed by DNAman software and synthesized by Sangon. Primer sequences used were: upstream (P1): 5′-ATGGCATTCCAG TTATGTCTC-3′ and downstream (P2): 5′-TCACATTACA GTGCTTTCCTTCTTGC-3′. Amplification of *Eg95* was conducted in a 20 μl mixture of 1 μl cDNA template, 2 μl 10X buffer, 0.5 μl of each primer, 0.5 μl of 10 mM dNTP, 0.5 μl *Taq* enzyme and 15.5 μl pure water. PCR reaction was carried out according to the following procedures: initial denaturation at 95°C for 6 min, then 30 consecutive cycles of denaturation at 95°C for 30 sec, annealing at 55°C for 30 sec, extension at 72°C for 2 min, and a final extension at 72°C for 10 min. The PCR products were detected by electrophoresis on a 1.2% agarose gel.

### Construction of recombinant plasmid pUCm-T/Eg95

After purification, PCR products were cloned into the pUCm-T vector. The ligation system was conducted in a 10 μl mixture of 2 μl PCR product, 0.5 μl pUCm-T vector, 1 μl 10X ligation buffer, 0.5 μl T4 DNA ligase and 6 μl de-ionized water. After ligation at 16°C for 16 h, the ligation product was transformed into *E. coli* DH5α competent cells. For clone screening, ampicillin LB plates were pre-treated with IPTG and X-Gal. After overnight incubation at 37°C, the positive white colonies were selected and incubated for PCR identification. The correct recombination plasmid was confirmed by sequencing and blasting.

### Tertiary structure prediction and analysis

Predictive analysis of the Eg95 protein tertiary structure was conducted by online server 3DLigandsite (http://www.sbg.bio.ic.ac.uk/~3dligandsite/) and the iterative-TASSER (I-TASSER). RasMol version 2.7.5.2 software was used to analyze different modes of the tertiary structure. The tertiary structure was displayed in the modes of Cartoon, Structure and Group.

### T- and B-combined epitope prediction and analysis

The B-cell epitope prediction software included DNAStar (V5.0) (http://www.dnastar.com) and the online prediction software IEDB (http://tools.immuneepitope.org/main/index.html). The T-cell epitope prediction software included SYFPEITHI (http://www.syfpeithi.de) and BIMAS (http://bimas.dcrt.nihgov/molbiothe/hla_bind/).

## Results

### Total RNAs were successfully extracted from Echinococcus protoscolex

**Figure 1:**
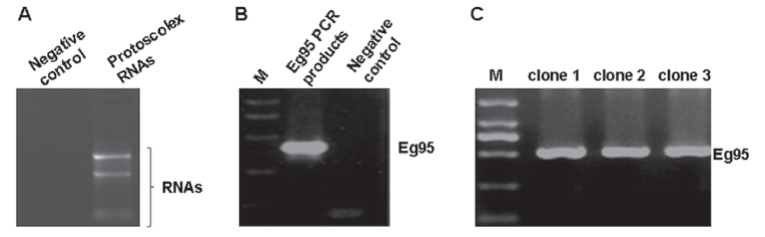
Recombinant plasmid pUCm-T/Eg95. (A) Total RNAs extracted from *Echinococcus* protoscolex were analyzed on 1.2% MOPS-formaldehyde denaturing gel. (B) Cloning of *Eg95* gene from *Echinococcus* cDNA. *Eg95* gene was amplified from *Echinococcus* cDNA by PCR assay. The PCR products were analyzed by agarose gel electrophoresis. (C) Confirmation of recombinant plasmid pUCm-T/Eg95. Correct construct of pUCm-T/Eg95 plasmid was identified by PCR assay. M, DL2000 DNA marker; lanes 1–3, PCR products of three white colonies.

In order to check the quality of the extracted RNA, total RNAs were analyzed on 1.2% MOPS-formaldehyde denaturing gel. The electrophoresis results are shown in Fig. 1A. On the gel, two bands were evident: The lower band was extremely smeared, while the upper band was much clearer. RNA absorbance of the upper band was measured at 260 and 280 nm. The value of A260/A280 was 1.81, indicating a good purity of RNA. This result suggests that total RNAs were extracted with good purity and could be used for subsequent analysis.

### Successful amplification of the Eg95 gene

A PCR assay was performed to clone the *Eg95* gene. Firstly the extracted RNAs were reversed transcribed into cDNA by RT-PCR. cDNA was subsequently used as a template to perform PCR. The PCR products were verified by gel electrophoresis. As shown in Fig. 1B, in the *Eg95* PCR products, a specific band of ~500 bp was identified while no such band was found in the negative control group. In theory, the gene length of *Eg95* was 471 bp. The length of this PCR fragment was close to that of the *Eg95* gene. This result indicates that a specific PCR fragment was amplified from the cDNA. However, whether the sequence of corresponds to the sequence of *Eg95* gene remains to be verified.

### Successful construction of the recombinant plasmid pUCm-T/Eg95

To evaluate the sequence of the amplified *Eg95* gene, we initially cloned the *Eg95* gene into the pUCm-T vector and the gene was screened using blue-white selection. A PCR assay was performed with the white clones to identify correctly constructed plasmids. Three white colonies were randomly selected for PCR analysis. As shown in Fig. 1C, all three colonies contained the *Eg95* gene fragment, demonstrating that the plasmid was successfully constructed.

Recombinant plasmid was sequenced and the sequence was compared to the *Eg95* gene sequence (Genebank accession no. HM345607) in BLAST. The alignment result showed that the sequence in the recombinant plasmid was correct.

Therefore, the *Eg95* gene was correctly amplified and the pUCm-T/Eg95 plasmid was successfully constructed.

### Tertiary structure prediction results of Eg95 protein

**Figure 2:**
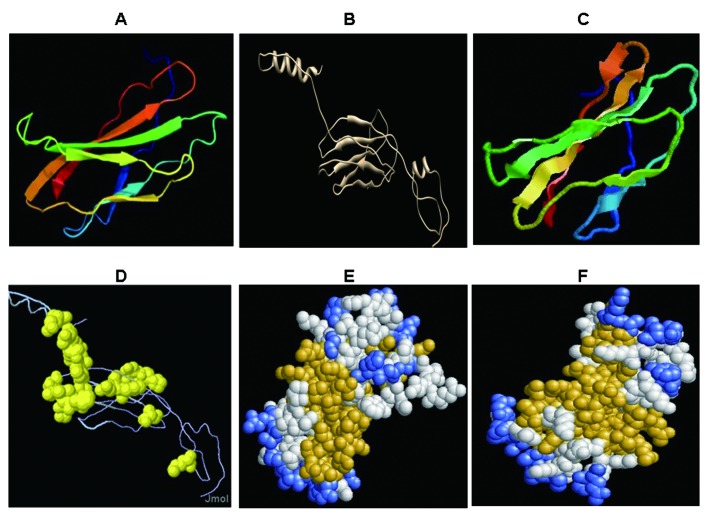
(A and B) Tertiary structure prediction results. Tertiary structure of the Eg95 predicted by 3DLigandsite. The structure of the protein was modeled using Phyre. The α helixes are shown by the curved lines and the β folds are shown by the laminated structures. (B) Tertiary structure of the Eg95 predicted by I-TASSER. In I-TASSER, the threading method and homology modeling method were used. (C–F) Structure was displayed in wireframe format. Different mode displays of tertiary structure for Eg95. The tertiary structure predicted by 3DLigandsite was further analyzed by RasMol version 2.7.5.2. The tertiary structure was displayed in (C) Cartoon, (D) Structure and (E and F) Group (E, front view and F, back view) modes. The laminated structures and the amino acids in the yellow area corresponded to the flexible regions of the protein.

In a previous study (13), we predicted the secondary structure of the Eg95 protein. The secondary structure indicated high flexibility and scalability of the Eg95 antigen. To determine the conformational structure of the Eg95 antigen, in the present study we predicted its tertiary structure. Fig. 2A was analyzed by the 3DLigandsite server. The tertiary structure of Eg95 comprised α helixes (shown by the curved lines) and β folds (shown by the laminated structures).

To gain a better understanding of its conformational structure, the tertiary structure of Eg95 was analyzed by I-TASSER. In I-TASSER, the threading method and homology modeling method were used. Subsequent to minor adjustments by the Swiss PDB viewer (SPDBV) software, the tertiary structure predicted by I-TASSER were identified (Fig. 2B). In the N- and C-terminal of the protein structure, a number of α helixes were identified. These structures showed that the Eg95 protein had good spatial flexibility and scalability. β folds similar to FN3 domain were predominantly evident in the middle. These β folds were connected by random coils and randomly folded into the conformational structure. Furthermore, the majority of these random coils contained >5 amino acid residues. Therefore, the β fold regions were the potential positions of the epitope.

### Mode display of Eg95 tertiary structure

Different modes were utilized to present the 3D structure of the protein. The tertiary structure predicted by 3DLigandsite was then analyzed by RasMol version 2.7.5.2. The tertiary structure was presented in Cartoon, Structure and Group modes, respectively (Fig. 2C–F). The Cartoon mode (Fig. 2C) was similar to that in Fig. 2A, with flexible regions in the laminated structures of the protein. In the Structure mode (Fig. 2D), the amino acids in the yellow area corresponded to the flexible region (flexible areas are easy to fold and to form antigen epitopes) in the secondary structure. The front and back view of the Group mode (aggregation mode) showed that the yellow region was gathered and distributed substantially at the surface of the structure (Fig. 2E and F). When an antibody binds an antigen, the antigen epitope needs to be fully exposed to facilitate better binding. Thus we speculate that the yellow area in this mode is the antigen epitope that potentially binds to the antibody.

### T- and B-combined epitope prediction results of Eg95 antigen

Based on the tertiary structure of Eg95 protein, we predicted the potential T- and B-combined epitopes of Eg95 antigen. The T-cell epitope was predicted by the SYFPEITHI and BIMAS software, while the B-cell epitope was predicted by software included DNAStar (V5.0) (http://www.dnastar.com) and the online prediction software IEDB. The prediction results are shown in Table I, with the T epitopes in bold. The overlapping regions of T- and B-cell epitopes formed the T- and B-combined epitopes. Four and six T- and B-combined epitopes were of human and mouse origin, respectively. Additionally, four T- and B-combined epitopes were of both human and mouse origin. Taken together, these results clearly demonstrate the specific regions of these T- and B-combined epitopes in the Eg95 antigen, further providing experimental data for epitope vaccine development.

## Discussion

In our previous study (13), we predicted the secondary structure of Eg95. In order to gain a better understanding of the spatial structure of the Eg95 protein, we predicted its tertiary structure in the present study. Based on the T/B epitope information obtained, as well as the tertiary structure, we analyzed the T- and B-combined epitopes of Eg95. The results demonstrate the information of the potential epitopes for Eg95 in detail.

Methods employed for tertiary structure prediction include the homology modeling, the threading and the ab initio prediction methods. The first two are also referred to as template-based methods. Based on the Swiss model, we conducted homology modeling with Eg95 protein. The similarity between these two methods was 16.39%. The Qmean Z-score was only −5.72, while the model similarity was <30%. Of note, the predicted Qmean Z-score was extremely low. Subsequently, the threading method was employed. The modeling accuracy via combination of the homology modeling and threading method was much higher than that of the Swiss Model and phyre. The three-dimensional structure is modeled based on the multiple-threading alignments of LOMETS and I-TASSER. I-TASSER usually provides the first and most reliable template parameters, with the C-score being the confidence assessment for the quality of prediction model. The calculation method is based on the parameters of thread form alignment and analog assembly parameters. The C scores usually range from −5 to 2, the higher the score a model yields, the higher the credibility. The TM score and root-mean-square deviation (RMSD) value are used to measure the similarity between the two structures. When the natural structure of a protein is unknown, they became crucial indicators of the quality of the modeling. The similarity between the two structures is measured by the TM value (14). Usually, a TM value of >0.5 shows a correct topological model, while that of <0.17 induces a random similarity model. Following submission of the Eg95 protein sequence (GenBank Accession no. ADJ94888.1) to the I-TASSER, a tertiary structure of Eg95 protein was predicted. In the present study the C-score was −2.17, TM-score was 0.46±0.15 and Exp.RMSD was 9.4±4.6. In general, the TM-score >0.5 is considered meaningful. The protein tertiary structure prediction results were based on the crystal diffraction model in the protein data bank library (?). Protein crystal diffraction models of multiple proteins are being explored and the database is constantly updated. Through bioinformatics prediction, only the tertiary structure that is closest to its natural conformation can be accessed. Subsequently, the conformational structure may be comprehensively analyzed via the phage display peptide library in combination with spatial epitope prediction.

T cells are able to recognize linear epitopes presented by antigen-presenting cells, while B cells are capable of identifying linear and/or conformational epitopes. Accordingly epitopes can be divided into T- and B-cell epitopes. The T- and B-combined epitopes are epitopes that can be co-recognized by both T and B cells. This co-recognization is of great significance for the effective removal of pathogens. Combined analysis and calculation of the antigenic T- and B-cell epitopes are able to screen out the overlapping areas in the two epitopes. In theory, epitopes that contain both T- and B-cell epitopes may have an advantage in immune response. The epitopes (antigenic determinants) are usually located on the surface of the molecule and have good hydrophilicity and ductility. If the specific regions of these epitopes were to be identified, the epitope fragments could be cloned into vectors to construct plasmids. Thus these plasmids potentially carry epitope fragments of *Echinococcus* that may induce a strong immune response (15–18). Yang *et al*(19) chemically synthesized T-cell epitope genes (sequences, 21–40 peptides) of protein VP1 of O-type foot-and-mouth disease virus (FMDV). The T-cell epitope, B-cell epitope (sequences, 141–160 peptides) and the β2-galactosidase genes were combined together to construct a recombinant plasmid. This recombinant plasmid was expressed in *E. coli* to generate the fusion protein vaccine. This vaccine was subsequently used to immunize animals. The results showed that the immune response induced by the peptide vaccine containing T-B joint epitopes was 7-fold higher than that by the peptide vaccine containing the B-cell epitope only. Additionally, the T-B joint epitope vaccine has been used in immune prevention against virus attacks. These data indicated that the T-B joint epitope vaccine simultaneously stimulated the humoral and cellular immune response and thus may have a strong protective effect. Therefore, the epitope vaccine designed based on the T- and B-combined epitope was an effective method in genetically engineered vaccine design, with potentially beneficial applications (20,21). In this study, we analyzed the human and mouse T- and B-combined epitopes of *Echinococcus*. In conclusion, future studies should focus on investigating the potential immune protective effects of these epitopes to develop epitope vaccines for mice immunization.

## Figures and Tables

**Table I:** The T-B cell epitope prediction of human and mouse.

Epitope orign	Epitope regions	Epitope sequences
Human	14–38	**VLAQEYKG**VGKGQGQQETPLRNHFN
	48–67	**RLSWEVQH**LSDLKGTDISLR
	92–113	GELKPS**TLYKMTVEA**VKAKKTI
	120–135	IETPRAGK**KESTVMTS**
Mouse	16–38	AQEYKGVGK**GQGQQETPL**RNHFN
	34–48	**RNHFNLTPV**GSQGIR
	50–72	SWEVQHLSDLKGTD**ISLRAVNPS**
	67–90	**RAVNPSDPL**VCKRQTAKFSDGQLA
	103–113	**LGFTVDIET**PR
	120–132	TV**MTSGSALTS**AI
Human and mouse	14–38	**VLAQEYKGVGKGQGQQETPLRNHFN**
	48–72	**RLSWEVQHLSDLKGTDISLR**AVNPS
	92–113	**GELKPSTLYKMTVEAVKAKKTI**
	120–135	**IETPRAGKKESTVMTS**

The T epitopes are shown in bold.

## References

[1] Eckert J, Conraths FJ, Tackmann K (2000). Echinococcosis: an emerging or re-emerging zoonosis?. Int J Parasitol.

[2] Nunnari G, Pinzone MR, Gruttadauria S (2012). Hepatic echinococcosis: clinical and therapeutic aspects. World J Gastroenterol.

[3] Jia HY, Ding JB, Fu YC (2008). Molecular characteristics of *Echinococcus* EgA31 vaccine. Chin J Parasit Dis.

[4] Ye EJ, Su LT, Jiang L (2000). Research progress of hydatid disease prevention and control. Chin J Parasitol and Parasitic Dis.

[5] Dalton JP, Mulcahy G (2001). Parasite vaccines - a reality?. Vet Parasitol.

[6] Lightowlers MW, Flisser A, Gauci CG, Heath DD, Jensen O, Rolfe R (2000). Vaccination against cysticercosis and hydatid disease. Parasitol Today.

[7] Kaba SA, McCoy ME, Doll TA (2012). Protective antibody and CD8(+) T-cell responses to the *Plasmodium falciparum* circumsporozoite protein induced by a nanoparticle vaccine. PLoS One.

[8] de Sousa EM, da Costa AC, Trentini MM, de Araújo Filho JA, Kipnis A, Junqueira-Kipnis AP (2012). Immunogenicity of a fusion protein containing immunodominant epitopes of Ag85C, MPT51, and HspX from *Mycobacterium tuberculosis* in mice and active TB infection. PLoS One.

[9] Zhang W, Li X, Lin Y, Tian D (2012). Identification of three H-2K(d) restricted CTL epitopes of NS4A and NS4B protein from Yellow fever 17D vaccine. J Virol Methods.

[10] Benlahrech A, Meiser A, Herath S (2012). Fragmentation of SIV-gag vaccine induces broader T cell responses. PLoS One.

[11] Lightowlers MW, Lawrence SB, Gauci CG, Young J, Ralston MJ, Maas D, Health DD (1996). Vaccination against hydatidosis using a defined recombinant antigen. Parasite Immunol.

[12] Lightowlers MW, Jensen O, Fernandez E (1999). Vaccination trials in Australia and Argentina confirm the effectiveness of the Eg95 hydatid vaccine in sheep. Int J Parasitol.

[13] Li YJ, Wang J, Zhao H (2011). Bioinformatics prediction of the *Echinococcus granulosus* Eg95 antigenic epitope. Chin J Zoonoses.

[14] Zhang Y, Skolnick J (2004). Scoring function for automated assessment of protein structure template quality. Proteins.

[15] Mustafa AS (2002). Development of new vaccines and diagnostic reagents against tuberculosis. Mol Immunol.

[16] Gulati S, Ngampasutadol J, Yamasaki R, McQuillen DP, Rice PA (2001). Strategies for mimicking Neisserial saccharide epitopes as vaccines. Int Rev Immunol.

[17] Berzofsky JA, Ahlers JD, Belyakov IM (2001). Strategies for designing and optimizing new generation vaccines. Nat Rev Immunol.

[18] Sette A, Fikes J (2003). Epitope-based vaccines: an update on epitope identification, vaccine design and delivery. Curr Opin Immunol.

[19] Yang ZJ, Lin Y, Li GJ (2000). Immune reactions in a guinea pig model induced by the anti-type O FMDV gene engineering vaccine that contains T-cell and B-cell epitopes. Fudan Univ J.

[20] Lin L, Tan B, Pantapalangkoor P (2013). *Acinetobacter baumannii* rOmpA vaccine dose alters immune polarization and immunodominant epitopes. Vaccine.

[21] Testa JS, Shetty V, Hafner J, Nickens Z, Kamal S, Sinnathamby G, Philip R (2012). MHC class I-presented T cell epitopes identified by immunoproteomics analysis are targets for a cross reactive influenza-specific T cell response. PLoS One.

